# PGRP negatively regulates NOD-mediated cytokine production in rainbow trout liver cells

**DOI:** 10.1038/srep39344

**Published:** 2016-12-19

**Authors:** Ju Hye Jang, Hyun Kim, Mi Jung Jang, Ju Hyun Cho

**Affiliations:** 1 Research Institute of Life Science, Gyeongsang National University, Jinju 52828, South Korea; 2 Division of Life Science, Gyeongsang National University, Jinju 52828, South Korea

## Abstract

Pattern-recognition receptors (PRRs) initiate innate immunity via pathogen recognition. Recent studies suggest that signalling pathways downstream of different PRRs and their crosstalk effectively control immune responses. However, the cross-regulation among PRRs and its effects have yet to be fully described in fish. Here, we examined the crosstalk between OmPGRP-L1, a long form of PGRP in rainbow trout, and other PRRs during pathogenic infections. OmPGRP-L1 expression was increased in RTH-149 cells by iE-DAP and MDP, which are agonists of NOD1 and NOD2, respectively. The silencing of NOD1 and NOD2 specifically inhibited the upregulation of OmPGRP-L1 expression induced by their cognate ligands. Suppression of RIP2 and NF-κB activation prevented the induction of OmPGRP-L1 expression. An *in silico* analysis and electrophoretic mobility shift assay revealed that the promoter of OmPGRP-L1 has NF-κB binding sites, suggesting that OmPGRP-L1 is produced through the NOD-RIP2-NF-κB signalling pathway. Loss-of-function and gain-of-function experiments indicated that OmPGRP-L1 downregulates the induction of NOD-mediated pro-inflammatory cytokine expression. Mechanistically, secreted OmPGRP-L1 inhibited the activation of the NOD-induced NF-κB pathway via downregulation of TAK1 and IκBα phosphorylation through A20 expression. Our data demonstrate that OmPGRP-L1 and NODs might play interdependent roles in the inflammatory response to bacterial infections in rainbow trout.

The innate immune system is the host’s first line of defence against infection^1^. The main role of this system is to recognise invading pathogens at an early stage and trigger an appropriate inflammatory response. The innate immune response relies on the recognition of evolutionarily conserved structures on pathogens, termed pathogen-associated molecular patterns (PAMPs), by a limited number of germline-encoded pattern recognition receptors (PRRs)^2^,^3^. After the recognition of PAMPs, PRRs induce several extracellular activation cascades, such as the complement pathway, and various intracellular signalling pathways, which lead to inflammatory responses. These inflammatory responses are essential for the effective clearance of pathogens; however, excessive responses can be dangerous to the host as exemplified by sepsis^4^. Therefore, these responses are tightly controlled by negative feedback loops and anti-inflammatory factors. In most cases, PRRs, such as Toll-like receptors (TLRs), nucleotide-binding oligomerisation domain-containing proteins (NODs) and peptidoglycan recognition proteins (PGRPs), recognise a given pathogen simultaneously or sequentially and then activate distinct and shared signalling pathways. This raises the possibility of crosstalk between the pathways as well as with other immunomodulatory signalling pathways generated by particular inflammatory environments. This interplay between signalling pathways eventually determines the specific immune response directed at clearing the pathogen^5^.

PGRPs are innate immune molecules that have been structurally conserved through the evolution of both invertebrate and vertebrate animals. PGRPs are antibacterial and recognise the bacterial cell-wall component peptidoglycan (PGN), a polymer of β-(1,4)-linked *N*-acetylglucosamine and *N*-acetylmuramic acid^6^. All PGRPs contain at least one conserved PGRP domain, which enables the interaction with bacterial PGN^7^. Recently, several PGRPs were identified in teleost fish, including zebrafish (*Danio rerio*)^8^, pufferfish (*Tetraodon nigroviridis*)^9^, rockfish (*Sebastes schlegeli*)^10^, large yellow croaker (*Pseudosciaena crocea*)^11^, grass carp (*Ctenopharyngodon idella*)^12^ and rainbow trout (*Oncorhynchus mykiss*)^13^. Fish PGRPs, like other vertebrate PGRPs, were originally believed to function as effector molecules rather than inducers of signalling cascades in antimicrobial defences because vertebrates have other PRRs that recognise PGN, such as TLR2 and NODs^14^. However, it was recently reported that fish PGRPs might also affect multiple intracellular pathways. In zebrafish, inhibition of Pglyrp5 expression in the developing embryo with small interfering RNA modified the expression of genes involved in several pathways, including immune and inflammatory responses, signalling pathways, transcription and metabolism^15^,^16^. Inhibition of Pglyrp5 increased the expression of TLR2, TLR3, mitogen-activated protein kinase (MAPK)-interacting serine/threonine kinase 2, the interleukin (IL)-17 receptor and nuclear factor (NF)-κB^16^. In a previous study, we showed that OmPGRP-L1, induced by bacterial stimulation, downregulated the expression of pro-inflammatory cytokines in the rainbow trout hepatoma cell line RTH-149^13^. Thus, fish PGRPs may directly or indirectly downregulate the immune response to bacteria to prevent a constant state of inflammation. Signalling pathways downstream of different PRRs and their crosstalk effectively control immune responses. However, the effects of crosstalk between fish PGRPs and other PRRs on immune responses remain ambiguous. Here, we examined the role of OmPGRP-L1 as a negative regulator of inflammatory responses in RTH-149 cells by assessing the crosstalk between OmPGRP-L1 and other PRRs with respect to pathogen recognition.

## Results

### iE-DAP and MDP induce OmPGRP-L1 expression in RTH-149 cells via NOD activation

**Figure 1:**
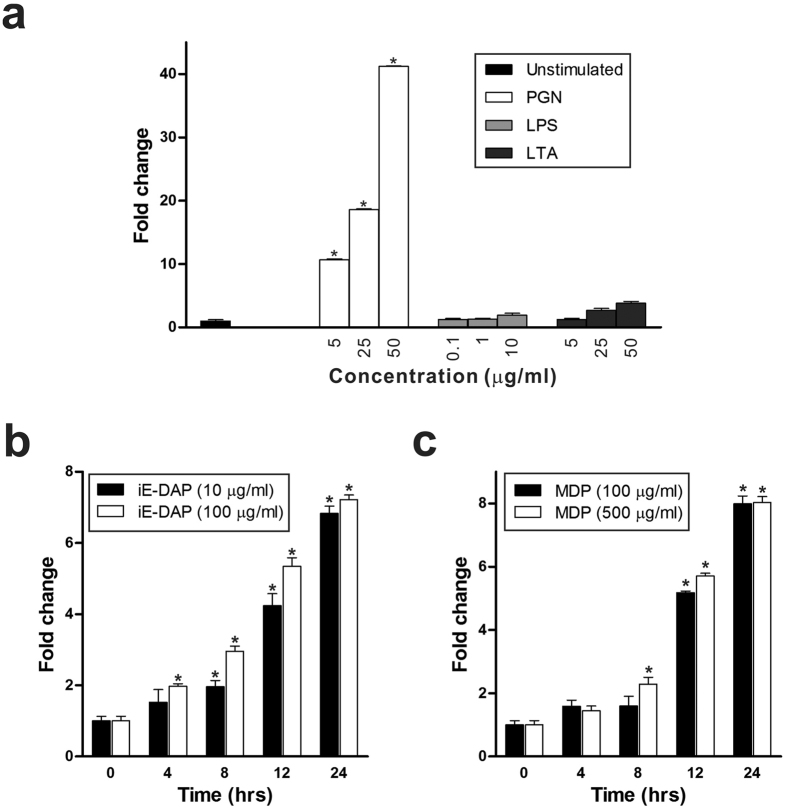
OmPGRP-L1 expression in RTH-149 cells in response to bacterial ligands and chemically synthesised iE-DAP and MDP. (**a**) RTH-149 cells were stimulated with PGN, LPS and LTA for 24 h at different doses, and OmPGRP-L1 expression was determined by qRT-PCR following stimulation. (**b**,**c**) RTH-149 cells were stimulated with iE-DAP and MDP at different doses, and OmPGRP-L1 expression was determined by qRT-PCR at different time points following stimulation. The expression level of OmPGRP-L1 was normalised by the β-actin level and presented as the relative fold compared with the non-treated control. The data in (**a**), (**b**) and (**c**) are shown as the mean ± SEM of three independent experiments performed in triplicate. *P < 0.05 when compared with control unstimulated cells.

Invading bacterial pathogens generally contain multiple PAMPs that are recognised by various PRRs. Upon PAMP recognition, PRRs induce signal transduction pathways, ultimately resulting in the activation of gene expression and synthesis of a broad range of molecules, including cytokines, chemokines, cell adhesion molecules and immunoreceptors^17^. In our previous study, we showed that bacterial stimulation increased OmPGRP-L1 expression in RTH-149 cells^13^. Therefore, to determine the PRR(s) involved in the induction of OmPGRP-L1 expression, we stimulated RTH-149 cells with various bacterial ligands, including PGN, lipopolysaccharide (LPS) and lipoteichoic acid (LTA). Among the tested bacterial ligands, PGN induced a dose-dependent increase in OmPGRP-L1 expression in RTH-149 cells, whereas no effect was observed in response to LPS and LTA stimulation (Fig. 1a). In addition, chemically synthesised γ-d-glutamyl-*meso*-diaminopimelic acid (iE-DAP) and muramyl dipeptide (MDP), the minimum essential structures responsible for the immunobiological activities of PGN, significantly induced a dose- and time-dependent increase in OmPGRP-L1 expression in RTH-149 cells (Fig. 1b and c).

**Figure 2:**
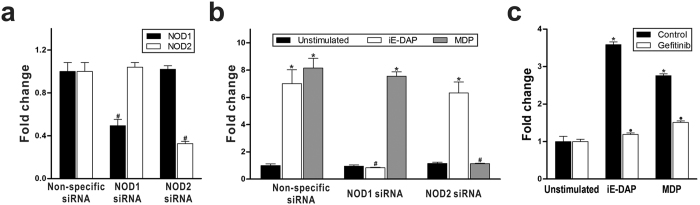
Involvement of NOD signalling pathways in OmPGRP-L1 expression in iE-DAP and MDP-stimulated RTH-149 cells. (**a**,**b**) RTH-149 cells were transfected with NOD1, NOD2 or non-specific siRNA for 48 h. The silencing of NOD1 and NOD2 was confirmed by qRT-PCR (**a**). At 48 h after transfection with each siRNA, cells (except control cells) were stimulated with iE-DAP (10 μg/ml) and MDP (100 μg/ml) for 24 h, and the expression of OmPGRP-L1 was analysed by qRT-PCR (**b**). (**c**) RTH-149 cells were pretreated with or without 10 μM gefitinib, an RIP2 inhibitor, for 30 min. After incubation, cells (except control cells) were stimulated with iE-DAP (100 μg/ml) and MDP (500 μg/ml) for 8 h, and the expression of OmPGRP-L1 was analysed by qRT-PCR. The data in (**a**), (**b**) and (**c**) are shown as the mean ± SEM of three independent experiments performed in triplicate. *P < 0.05, control unstimulated versus iE-DAP- or MDP-stimulated cells; ^#^*P* < 0.05, non-specific siRNA versus NOD1 or NOD2 siRNA; ^●^*P* < 0.05, non-pretreated versus gefitinib-pretreated cells.

iE-DAP and MDP possess NOD1- and NOD2-stimulatory activity, respectively^18^,^19^; therefore, we hypothesised that OmPGRP-L1 expression was induced via NOD activation in iE-DAP- and MDP-stimulated RTH-149 cells. To clarify the signalling pathway of cellular activation by iE-DAP and MDP, we utilised RNA interference assays targeting NOD1 and NOD2. NOD1 and NOD2 mRNA levels were suppressed by approximately 51.51% and 67.36%, respectively, using specific siRNAs in RTH-149 cells (Fig. 2a). As shown in Fig. 2b, the upregulated expression of OmPGRP-L1 induced by iE-DAP and MDP was markedly inhibited in NOD1- and NOD2-silenced RTH-149 cells, respectively. Several studies showed that NOD1 and NOD2 signalling activated receptor-interacting serine/threonine-protein kinase 2 (RIP2, also known as RICK)^20^,^21^,^22^. Therefore, we examined whether an RIP2 inhibitor (gefitinib) could attenuate the upregulation of OmPGRP-L1 expression induced by iE-DAP and MDP. Consistent with the previous reports, the RIP2 inhibitor markedly blocked the induction of OmPGRP-L1 expression induced by iE-DAP and MDP in RTH-149 cells (Fig. 2c). These results clearly indicated that iE-DAP and MDP regulate OmPGRP-L1 expression via the NOD1 and NOD2 signalling pathways, respectively.

### iE-DAP- and MDP-induced OmPGRP-L1 expression in RTH-149 cells requires NF-κB

**Figure 3:**
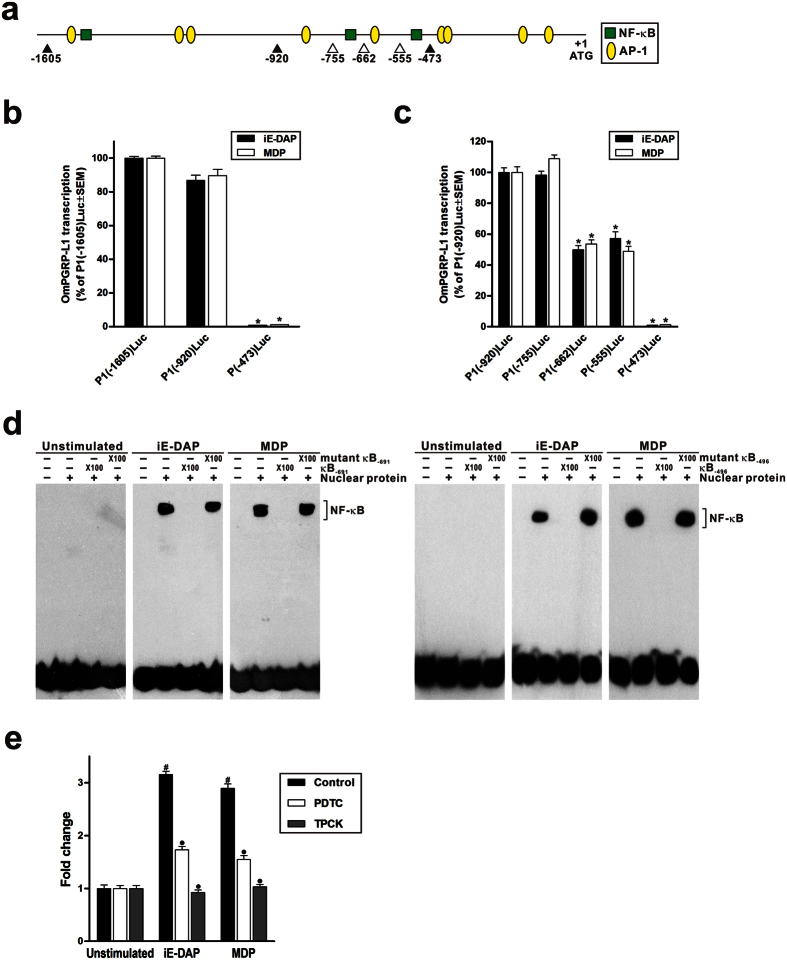
Involvement of NF-κB in OmPGRP-L1 expression in iE-DAP and MDP-stimulated RTH-149 cells. (**a**) Summary of the potential NF-κB and AP-1 binding sites. The closed and open triangles and the numbers below indicate the 5′ ends of OmPGRP-L1 gene promoter fragments that were cloned upstream of the luciferase gene. (**b**,**c**) RTH-149 cells were transfected with the indicated OmPGRP-L1 gene promoter-luciferase plasmids and stimulated with iE-DAP (10 μg/ml) and MDP (100 μg/ml) for 24 h. OmPGRP-L1 transcription was measured as luciferase activity. (**d**) RTH-149 cells (except control cells) were stimulated with iE-DAP (10 μg/ml) and MDP (100 μg/ml) for 24 h. Nuclear extracts from RTH-149 cells were incubated with biotin-labelled oligonucleotides homologous to the OmPGRP-L1 promoter containing κB_−691_ or κB_−496_ sites. For a subset of experiments, nuclear extracts were preincubated with an increasing amount of unlabelled specific or non-specific oligonucleotides prior the addition of the biotin-labelled oligonucleotide probe. (**e**) RTH-149 cells were pretreated with or without 25 μM of NF-κB inhibitors (PDTC and TPCK) for 30 min. After incubation, cells (except control cells) were stimulated with iE-DAP (100 μg/ml) and MDP (500 μg/ml) for 8 h, and OmPGRP-L1 expression was analysed by qRT-PCR. The data in (**b**), (**c**) and (**e**) are shown as the mean ± SEM of three independent experiments performed in triplicate. *P < 0.05, compared with P1(-1605)Luc or P1(-920)Luc; ^#^*P* < 0.05, control unstimulated versus iE-DAP- or MDP-stimulated cells; ^●^*P* < 0.05, non-pretreated versus NF-κB inhibitor-pretreated cells.

To identify the regulatory elements and transcription factors involved in the regulation of OmPGRP-L1 expression, we first isolated the 5′-flanking region of the OmPGRP-L1 gene using thermal asymmetric interlaced PCR (TAIL-PCR)^23^ and inspected this region for the presence of potential transcription factor binding sites. The *in silico* analysis using TRANSFAC and CONSITE data revealed several putative binding sites for NF-κB and activator protein 1 (AP-1) on the OmPGRP-L1 gene promoter at different sites (Supplementary Fig. S1 and Fig. 3a). It is well documented that the inflammatory response initiated by NODs induces the expression of pro-inflammatory cytokines, chemokines and antimicrobial molecules by activating the transcription factors NF-κB and AP-1^3^,^24^. To identify promoter regions that regulate OmPGRP-L1 expression in iE-DAP- and MDP-stimulated RTH-149 cells, we constructed OmPGRP-L1 gene promoter-luciferase plasmids. The plasmids contained a 1,605-bp [P1(-1605)Luc], 920-bp [P1(-920)Luc] or 473-bp [P1(-473)Luc] region upstream of the start codon. Next, we transfected these plasmids into RTH-149 cells. After stimulating the cells with iE-DAP and MDP, OmPGRP-L1 transcription was determined by measuring luciferase activity. P1(-1605)Luc and P1(-920)Luc were highly induced by both iE-DAP and MDP, whereas induction was abrogated using the 473-bp promoter (Fig. 3b). These results indicate that the regulatory elements necessary for the induction of OmPGRP-L1 transcription in RTH-149 cells stimulated with iE-DAP and MDP are located within the 473–920-bp region upstream of the start codon. To narrow down the region of the promoter required for the induction of OmPGRP-L1 expression in RTH-149 cells stimulated with iE-DAP and MDP, we examined the expression of OmPGRP-L1 gene promoter-luciferase plasmids using serial deletions within the 473–920-bp region upstream of the start codon (Fig. 3c). P1(-755)Luc was as active as P1(-920)Luc in RTH-149 cells stimulated with iE-DAP and MDP. In contrast, the promoter activity decreased when the region between −755 and −662 bp was deleted and completely abrogated after deletion of the region between −555 and −473 bp. These results indicate that these regions, which contain NF-κB binding sites at −691 bp (κB_−691_ site) and −496 bp (κB_−496_ site), are required for maximal OmPGRP-L1 expression in RTH-149 cells stimulated with iE-DAP and MDP.

NF-κB is a key transcription factor involved in the regulation of immune responses^25^. Therefore, we examined whether NF-κB was involved in the regulation of OmPGRP-L1 expression. Oligonucleotides spanning the κB_−691_ and κB_−496_ sites were used to determine the binding of proteins in an electrophoretic mobility shift assay (EMSA) (Fig. 3d). Nuclear extracts from unstimulated RTH-149 cells did not contain proteins that bind κB_−691_ and κB_−496_ sites. However, nuclear extracts from RTH-149 cells stimulated with iE-DAP and MDP contained proteins that bound to both κB_−691_ and κB_−496_ sites. The specificity of binding of nuclear proteins from iE-DAP- and MDP-stimulated RTH-149 cells to the κB_−691_ and κB_−496_ sequences was confirmed using excess unlabelled specific and non-specific oligonucleotides. The binding of nuclear proteins to both the κB_−691_ and κB_−496_ sequences was inhibited by an excess of unlabelled specific oligonucleotides for the κB_−691_ and κB_−496_ sites but not by non-specific oligonucleotides containing several mutated nucleotides within the κB_−691_ and κB_−496_ sites. These results show that nuclear proteins from RTH-149 cells stimulated with iE-DAP and MDP specifically bind to the κB_−691_ and κB_−496_ sites on the OmPGRP-L1 gene promoter. In addition, the NF-κB inhibitors ammonium pyrrolidinedithiocarbamate (PDTC) and *N*-p-tosyl-L-phenylalanine chloromethyl ketone (TPCK) markedly prevented the induction of OmPGRP-L1 expression induced by iE-DAP and MDP in RTH-149 cells (Fig. 3e). Thus, the transcription factor NF-κB is absolutely required for the induction of OmPGRP-L1 expression in RTH-149 cells stimulated with iE-DAP and MDP.

### OmPGRP-L1 opposes the effect of NODs on iE-DAP- and MDP-induced activation of pro-inflammatory cytokines

**Figure 4:**
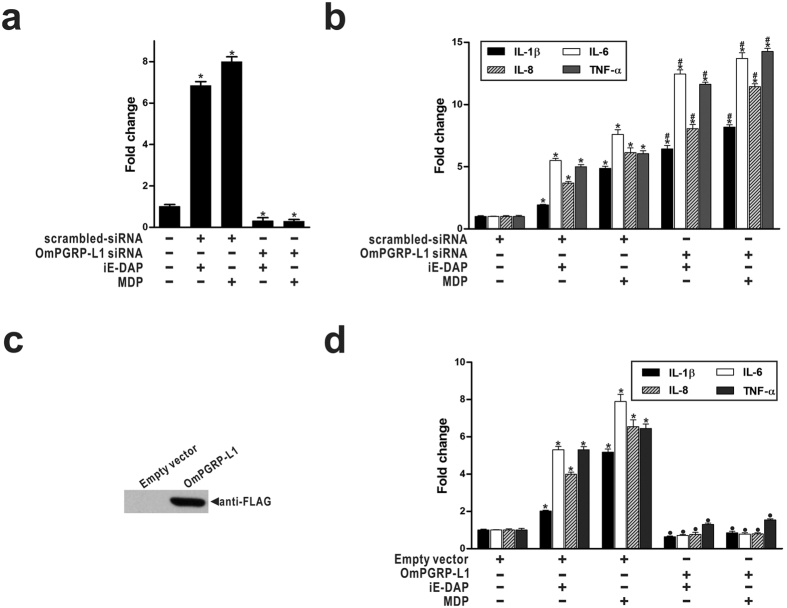
The effect of OmPGRP-L1 silencing or overexpression on the expression of pro-inflammatory cytokines in iE-DAP- and MDP-stimulated RTH-149 cells. (**a,b**) RTH-149 cells were transfected with OmPGRP-L1 siRNA or scrambled-siRNA. After 48 h, cells (except control cells) were stimulated with iE-DAP (10 μg/ml) and MDP (100 μg/ml) for 24 h, and the expression of OmPGRP-L1 (**a**) and pro-inflammatory cytokines (**b**) was analysed by qRT-PCR. (**c,d**) RTH-149 cells were transfected with pcDNA3.1-OmPGRP-L1-FLAG or an empty pcDNA3.1 vector. OmPGRP-L1 overexpression was confirmed by western blotting with a mouse monoclonal anti-FLAG antibody (**c**). At 48 h after transfection, cells (except control cells) were stimulated with iE-DAP (10 μg/ml) and MDP (100 μg/ml) for 24 h, and the expression of pro-inflammatory cytokines was analysed by qRT-PCR (**d**). The data in (**a**), (**b**) and (**d**) are shown as the mean ± SEM of three independent experiments performed in triplicate. *P < 0.05, control unstimulated versus iE-DAP- or MDP-stimulated cells; ^#^*P* < 0.05, scrambled-siRNA versus OmPGRP-L1 siRNA; ^●^*P* < 0.05, empty vector versus OmPGRP-L1.

NOD1 and NOD2 signalling contributes to host defence via the production of pro-inflammatory cytokines and antimicrobial molecules in rainbow trout^22^,^26^. In contrast, OmPGRP-L1 exerts an anti-inflammatory function by downregulating the expression of pro-inflammatory cytokines in *Edwardsiella tarda*-stimulated RTH-149 cells^13^, suggesting that OmPGRP-L1 acts as a negative regulator during inflammatory responses to bacterial infections. We examined the effect of OmPGRP-L1 on NOD-mediated pro-inflammatory cytokine production in RTH-149 cells. We first evaluated the effect of decreased OmPGRP-L1 expression on the expression of pro-inflammatory cytokines (IL-1β, IL-6, IL-8 and TNF-α) using RNA interference in cells stimulated with iE-DAP and MDP for 24 h. The iE-DAP- and MDP-induced increase in OmPGRP-L1 expression was mitigated by transfecting RTH-149 cells with siRNA targeted to OmPGRP-L1 (Fig. 4a). Transfection with scrambled siRNA did not influence OmPGRP-L1 expression; thus, the gene silencing specificity was confirmed. The expression of IL-1β, IL-6, IL-8 and TNF-α in RTH-149 cells transfected with scrambled siRNA was increased after stimulation with iE-DAP (1.92-, 5.5-, 3.69- and 5-fold, respectively) and MDP (4.87-, 7.59-, 6.14- and 6.05-fold, respectively) (Fig. 4b). In iE-DAP- and MDP-stimulated cells, the silencing of OmPGRP-L1 significantly increased the expression of IL-1β (6.43- and 8.19-fold, respectively), IL-6 (12.45- and 13.7-fold, respectively), IL-8 (8.06- and 11.43-fold, respectively) and TNF-α (11.62- and 14.28-fold, respectively) (Fig. 4b).

Next, we evaluated the effect of OmPGRP-L1 overexpression on the expression of pro-inflammatory cytokines. RTH-149 cells were transfected with an OmPGRP-L1 expression vector (pcDNA3.1-OmPGRP-L1-FLAG) or empty pcDNA3.1 vector for 48 h. OmPGRP-L1 overexpression was confirmed by western blotting with an anti-FLAG antibody (Fig. 4c). The expression of IL-1β, IL-6, IL-8 and TNF-α in RTH-149 cells transfected with the empty vector was increased after stimulation with iE-DAP (2.01-, 5.3-, 3.99- and 5.3-fold, respectively) and MDP (5.17-, 7.89-, 6.54- and 6.45-fold, respectively) (Fig. 4d). In iE-DAP- and MDP-stimulated cells, OmPGRP-L1 overexpression inhibited IL-1β (0.63- and 0.85-fold, respectively), IL-6 (0.71- and 0.77-fold, respectively), IL-8 (0.76- and 0.78-fold, respectively) and TNF-α (1.3- and 1.54-fold, respectively) expression (Fig. 4d). Overall, these data indicated that OmPGRP-L1 downregulates the induction of NOD-mediated pro-inflammatory cytokine expression in RTH-149 cells.

### OmPGRP-L1 inhibits the activation of the NOD-induced NF-κB pathway

**Figure 5:**
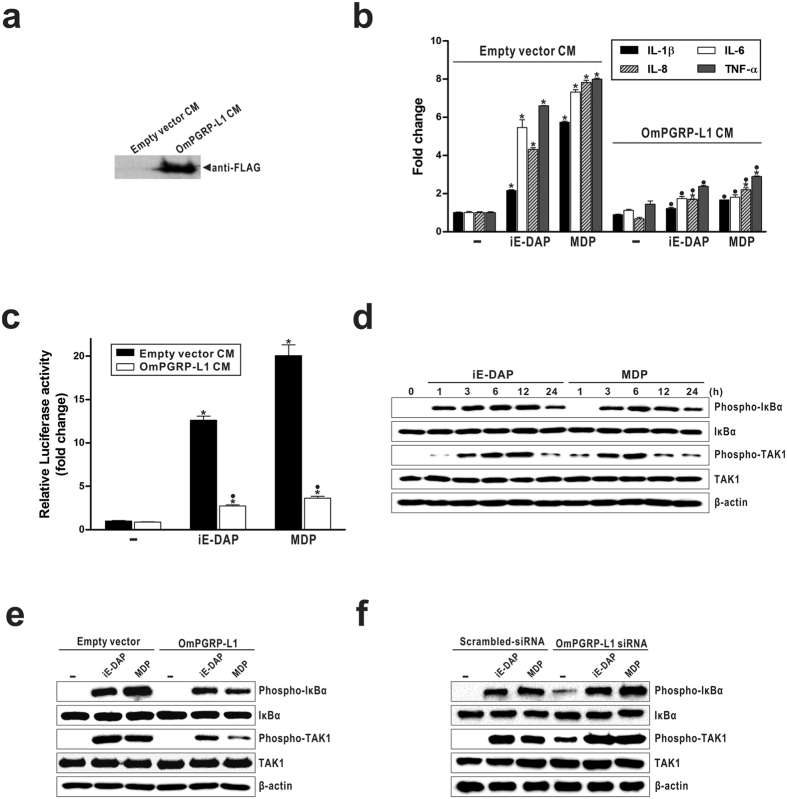
The effect of secreted OmPGRP-L1 on the NOD-induced NF-κB pathway in iE-DAP- and MDP-stimulated RTH-149 cells. (**a**) The secretion of OmPGRP-L1-FLAG recombinant protein in the conditioned media was confirmed by western blotting with a mouse monoclonal anti-FLAG antibody. (**b**) RTH-149 cells were cultured in 6-well plates, and the cell culture medium was replaced with the conditioned media containing the secreted OmPGRP-L1-FLAG recombinant protein (OmPGRP-L1 CM) or empty vector CM. Next, cells were stimulated with 10 μg/ml iE-DAP and 100 μg/ml MDP for 24 h, and the expression of pro-inflammatory cytokines was analysed by qRT-PCR. (**c**) RTH-149 cells were co-transfected with pNF-κB-Luc and pRL-TK vectors. At 48 h after transfection, the cell culture medium was replaced with conditioned media. Next, cells were stimulated with 10 μg/ml iE-DAP and 100 μg/ml MDP for 24 h, and NF-κB activity was measured as described in the Methods. (**d**) RTH-149 cells were stimulated with iE-DAP (10 μg/ml) and MDP (100 μg/ml) for the indicated times. Next, the phospho- and total forms of IκBα and TAK1 were assessed by western blotting. (**e**,**f**) RTH-149 cells were transfected with pcDNA3.1-OmPGRP-L1-FLAG, empty pcDNA3.1 vector, OmPGRP-L1 siRNA or scrambled-siRNA. At 48 h after transfection, cells (except control cells) were stimulated with iE-DAP (10 μg/ml) and MDP (100 μg/ml) for 6 h. Next, the phospho- and total forms of IκBα and TAK1 were assessed by western blotting. The data in (**b**) and (**c**) are shown as the mean ± SEM of three independent experiments performed in triplicate. *P < 0.05, control unstimulated versus iE-DAP- or MDP-stimulated cells; ^●^*P* < 0.05, empty vector CM versus OmPGRP-L1 CM.

In a previous study, we showed that OmPGRP-L1 overexpression in RTH-149 cells inhibits NF-κB activity with or without bacterial stimulation^13^. However, it is unclear how OmPGRP-L1, which is a secreted protein with a signal peptide, inhibits NF-κB activity. To clarify this mechanism, we first examined whether OmPGRP-L1 is a secreted protein. The pcDNA3.1-OmPGRP-L1-FLAG vector or empty pcDNA3.1 vector were transiently transfected into RTH-149 cells, and the conditioned medium was harvested (OmPGRP-L1 CM and empty vector CM, respectively). The secreted OmPGRP-L1-FLAG recombinant protein was successfully expressed by RTH-149 cells and could be detected in OmPGRP-L1 CM (Fig. 5a). We next performed media exchange experiments to determine whether secreted OmPGRP-L1 inhibited the production of NOD-mediated pro-inflammatory cytokines in RTH-149 cells. We cultured RTH-149 cells in 6-well plates and replaced the culture media with the conditioned media containing the secreted recombinant OmPGRP-L1-FLAG recombinant protein (OmPGRP-L1 CM) or empty vector CM. The expression of IL-1β, IL-6, IL-8 and TNF-α in RTH-149 cells cultured with empty vector CM was increased after stimulation with iE-DAP (2.17-, 5.45-, 4.31- and 6.6-fold, respectively) and MDP (5.74-, 7.32-, 7.82- and 7.99-fold, respectively) compared with the control unstimulated cells (Fig. 5b). On the other hand, the addition of OmPGRP-L1 CM in iE-DAP- and MDP-stimulated cells inhibited the expression of IL-1β (1.22- and 1.66-fold, respectively), IL-6 (1.74- and 1.8-fold, respectively), IL-8 (1.7- and 2.2-fold, respectively) and TNF-α (2.37- and 2.89-fold, respectively) (Fig. 5b). The addition of OmPGRP-L1 CM also reduced NF-κB activity by ~78.4% and ~81.9% in iE-DAP- and MDP-stimulated RTH-149 cells, respectively (Fig. 5c).

**Figure 6:**
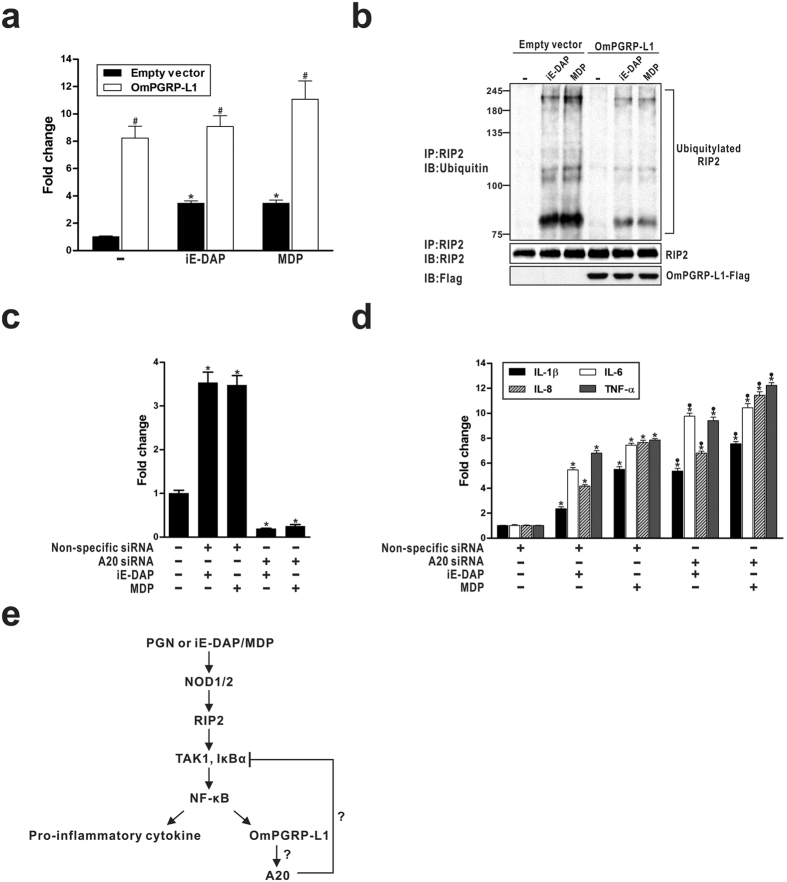
Involvement of A20 in OmPGRP-L1-mediated negative regulation of NOD signalling pathways in iE-DAP- and MDP-stimulated RTH-149 cells. (**a,b**) RTH-149 cells were transfected with pcDNA3.1-OmPGRP-L1-FLAG or an empty pcDNA3.1 vector. At 48 h after transfection, cells (except control cells) were stimulated with iE-DAP (10 μg/ml) and MDP (100 μg/ml) for 24 h. A20 expression was analysed by qRT-PCR (**a**). Cells were lysed in RIPA buffer and were immunoprecipitated with anti-RIP2 antibody (**b**). The immunoprecipitated polyubiquitylated proteins were subjected to SDS-PAGE, and western blotting was performed using an anti-ubiquitin antibody (upper blot). The immunoprecipitates were also probed with anti-RIP2 antibody to ensure that equivalent amounts of RIP2 were immunoprecipitated (middle blot). The total cell lysates were probed with a mouse monoclonal anti-FLAG antibody to confirm OmPGRP-L1 overexpression (lower blot). (**c,d**) RTH-149 cells were transfected with A20 siRNA or non-specific siRNA. After 48 h, cells (except control cells) were stimulated with iE-DAP (10 μg/ml) and MDP (100 μg/ml) for 24 h, and the expression of A20 (**c**) and pro-inflammatory cytokines (**d**) was analysed by qRT-PCR. (**e**) Schematic diagram of the crosstalk between NODs and OmPGRP-L1 signalling in PGN- or PGN fragment-induced inflammatory responses in RTH-149 cells. The diagram is based on our findings from this study. The data in (**a**), (**c**) and (**d**) are shown as the mean ± SEM of three independent experiments performed in triplicate. *P < 0.05, control unstimulated versus iE-DAP- or MDP-stimulated cells; ^#^*P* < 0.05, empty vector versus OmPGRP-L1; ^●^*P* < 0.05, non-specific siRNA versus A20 siRNA.

In NOD signalling, the phosphorylation of transforming growth factor-β-activated kinase (TAK) 1 and inhibitor of κBα (IκBα) are key upstream signals for NF-κB activation^27^. Thus, we examined whether OmPGRP-L1 inhibited the NOD-mediated phosphorylation of TAK1 and IκBα using western blotting. As shown in Fig. 5d, stimulation with iE-DAP and MDP enhanced the phosphorylation of TAK1 and IκBα in RTH-149 cells at 12 h and 6 h after stimulation, respectively. This phosphorylation was significantly blocked by OmPGRP-L1 overexpression (Fig. 5e). In contrast, the silencing of OmPGRP-L1 significantly increased the phosphorylation of TAK1 and IκBα in iE-DAP- and MDP-stimulated cells (Fig. 5f). Recent studies demonstrated that K63-linked regulatory ubiquitylation of RIP2 was essential for the recruitment of TAK1 in NOD signalling pathways^28^,^29^. A20, a deubiquitinase that removes K63-linked polyubiquitin chains, dampens NOD-induced NF-κB activation^28^,^30^. Thus, we hypothesised that OmPGRP-L1 negatively regulates NOD signalling by inducing A20 expression. To test this hypothesis, we investigated the effect of OmPGRP-L1 overexpression on the expression of A20 in RTH-149 cells. When overexpressed in RTH-149 cells, OmPGRP-L1 increased A20 expression with or without iE-DAP- and MDP-stimulation (Fig. 6a). After stimulation with iE-DAP and MDP for 24 h, the expression of A20 in RTH-149 cells transfected with empty vector was induced by 3.47- and 3.46-fold, respectively, compared with the control unstimulated cells transfected with empty vector. In contrast, OmPGRP-L1 overexpression significantly increased A20 expression in both iE-DAP- and MDP-stimulated cells (9.08-fold and 11.07-fold, respectively) and unstimulated cells (8.24-fold). We then asked whether OmPGRP-L1-induced A20 could regulate RIP2’s signalling activity by regulating RIP2 ubiquitylation in iE-DAP- and MDP-stimulated cells. To measure the status of RIP2, we treated cells (transfected with an OmPGRP-L1 expression vector or empty vector) with iE-DAP and MDP, immunoprecipitated RIP2, and tested the ubiquitylation status of RIP2 by immunoblotting for ubiquitin. These experiments revealed that overexpression of OmPGRP-L1 reduced the amount and size of polyubiquitylated RIP2 in iE-DAP- and MDP-stimulated cells (Fig. 6b). In addition, the silencing of A20 using an A20-specific siRNA, which was confirmed by qRT-PCR (Fig. 6c), significantly increased the expression of pro-inflammatory cytokines in iE-DAP- and MDP-stimulated RTH-149 cells (Fig. 6d). Overall, these data indicated that secreted OmPGRP-L1 negatively regulates the activation of the NOD-induced NF-κB pathway via inhibition of the NOD-mediated phosphorylation of TAK1 and IκBα through A20 expression (Fig. 6e).

## Discussion

Over the past few years, the mechanisms underlying the ligand specificity, signalling pathways and subcellular localisation of PRRs, such as TLRs, NODs and PGRPs, have been extensively characterised in vertebrates. Given the highly complicated and variable repertoire of PAMPs that are recognised by PRRs and the relatively independent, but partially overlapping, signalling pathways they induce, distinct PRRs must cooperate to generate the most appropriate effector immune responses^31^. Recently, it was reported that fish PGRPs might affect multiple intracellular pathways^15^,^16^, suggesting that crosstalk between fish PGRPs and other PRRs modulate their functions. However, the cross-regulation of fish PGRPs and other PRRs as well as its downstream effects during pathogenic infections have not been thoroughly described. Our present study is the first to demonstrate that PGRP and NODs, two different PRRs, might play interdependent roles in the inflammatory response to bacterial infections in rainbow trout.

A previous link between NODs, which are cytosolic proteins involved in the intracellular recognition of microbes and their products^32^, and PGRP showed that bacteria and their products increased the expression of mammalian PGLYRP-3 and PGLYRP-4 in keratinocytes^33^, fibroblasts and oral epithelial cells^34^ via NOD activation. In this study, we determined that OmPGRP-L1 expression was upregulated in RTH-149 cells stimulated with PGN and PGN fragments, iE-DAP and MDP, which are agonists of NOD1 and NOD2, respectively (Fig. 1). The silencing of NOD1 and NOD2 specifically inhibited the upregulation of OmPGRP-L1 expression induced by their cognate ligands (Fig. 2b). The suppression of RIP2 and NF-κB activation prevented iE-DAP- and MDP-induced OmPGRP-L1 expression (Figs 2c and 3e). An *in silico* analysis and EMSA revealed that the OmPGRP-L1 promoter contains NF-κB binding sites (Fig. 3a–d). Overall, these results clearly indicated that OmPGRP-L1 was produced through the NOD-RIP2-NF-κB signalling pathway, thus suggesting a reciprocal regulation of expression between NODs and OmPGRP-L1.

Our results demonstrate that OmPGRP-L1 modulates inflammation and cross-regulates with NODs to protect the host from excessive inflammation. NOD signalling contributes to host defence via the production of pro-inflammatory cytokines and antimicrobial molecules in rainbow trout^22^,^26^. In contrast, OmPGRP-L1 downregulates the induction of NOD-mediated pro-inflammatory cytokine expression in RTH-149 cells, suggesting that OmPGRP-L1 is a negative regulator of NOD signalling (Fig. 4). In general, negative regulators of NOD signalling may inhibit NOD1 and/or NOD2 interactions with other molecules in the pathway. For example, NOD signalling was inhibited by Centaurin β-1 (CENTβ1), a GTPase-activating protein from the ADP-ribosylation family that colocalises with NOD1 and NOD2 in the cytoplasm of intestinal epithelial cells. CENTβ1 overexpression inhibited NOD1- and NOD2-dependent NF-κB signalling^35^. In our previous study, we showed that OmPGRP-L1 overexpression in RTH-149 cells inhibited NF-κB activity^13^. However, unlike other negative regulators of NOD signalling, such as CENTβ1, OmPGRP-L1 is not a cytosolic protein but rather a secreted protein (Fig. 5a), which precludes the possibility of direct interaction with other molecules in the NOD signalling pathway. In this study, we confirmed that secreted OmPGRP-L1 inhibited the production of NOD-mediated pro-inflammatory cytokines by reducing NF-κB activity in RTH-149 cells (Fig. 5b and c). Saha *et al*. recently reported that mouse PGLYRP-2, which is also a secreted protein, functions as an alarmin in a PGN-induced arthritis model^36^. This raises the possibility of a dedicated cell-surface receptor for PGLYRP-2^37^. It is postulated that OmPGRP-L1, similar to PGLYRP-2, functions as a cytokine-like molecule. OmPGRP-L1 may modulate NOD signalling through the induction of other regulator protein(s), such as phosphatases and kinases, ubiquitin-related proteins, transcription factors and epigenetic molecules, which contribute to distinct steps of the NOD signalling cascade. For example, the deubiquitylating enzyme A20 is induced by IL-17 and mediates negative feedback by targeting TNF receptor-associated factor 6 (TRAF6) and thus reduces NF-κB and MAPK signalling^38^. In this study, OmPGRP-L1 overexpression inhibited the NOD-mediated phosphorylation of TAK1 and IκBα (Fig. 5e and 5f) and increased the expression of A20 with or without iE-DAP and MDP stimulation (Fig. 6a). In addition, OmPGRP-L1-induced A20 restricts NOD-mediated signalling to NF-κB by interfering with NOD-induced ubiquitylation of RIP2 (Fig. 6b). Consistently, the silencing of A20 led to enhanced pro-inflammatory cytokine expression in iE-DAP- and MDP-stimulated RTH-149 cells (Fig. 6d). Additional studies are required to dissect the specific pathways involved; however, our data suggest that OmPGRP-L1 negatively regulates the activation of the NOD-induced NF-κB pathway, possibly via the inhibition of the NOD-mediated phosphorylation of TAK1 and IκBα through A20 expression (Fig. 6e).

Receptor crosstalk in the innate immune system is crucial for the coordination of microorganism-sensing signals and an appropriate immune response^39^. The outcome of the cross-regulation between different PRRs can be synergistic or antagonistic, i.e. amplify or impair the inflammatory response. Two characteristic examples are the cooperation of TLR2 with C-type lectin dectin 1 (also known as CLEC7A) to stimulate antifungal immunity^40^ and the homeostatic suppression of TLR-induced pro-inflammatory responses by the glucocorticoid and adenosine receptors^41^,^42^. In the cross-regulation between PGRP and NOD, it has been reported that mouse PGLYRP-2 cooperates with NOD2 to activate pro-inflammatory genes in a PGN-induced arthritis model^36^. However, the role of PGLYRP-2 in arthritis is unique because other mouse PGLYRPs, including PGLYRP-1, -3 and -4, do not have similar pro-inflammatory effects. Indeed, PGLYRP-1^−/−^, PGLYRP-3^−/−^ and PGLYRP-4^−/−^ mice all had higher MDP-induced activation of pro-inflammatory genes than wild-type mice^36^; thus, these proteins may have an anti-inflammatory effect. In the current study, we clearly showed that OmPGRP-L1 limited NOD-induced inflammatory responses in a feedback manner in RTH-149 cells. These results suggest that OmPGRP-L1 modifies the primary responses of NODs to their agonists, which adds another level of complexity to the regulation of innate immunity.

In summary, we demonstrated that PGN and PGN fragments activated NODs and induced OmPGRP-L1 expression through the NOD-RIP2-NF-κB signalling pathway. Secreted OmPGRP-L1 inhibited NOD-induced inflammatory responses via the downregulation of TAK1 and IκBα phosphorylation through A20 expression. Although additional *in vivo* studies are required to clarify the current *in vitro* data, our findings indicate that OmPGRP-L1, in conjunction with NODs, might help maintain a fine balance between protective immunity and inflammatory pathology in rainbow trout.

## Methods

### Reagents

*Staphylococcus aureus* PGN and LTA, *Escherichia coli* 0111:B4 LPS, TPCK and PDTC were purchased from Sigma-Aldrich (USA). iE-DAP, MDP and gefitinib were purchased from InvivoGen (USA). The antibodies against TAK1, phosphorylated TAK1 (Thr184/187), phosphorylated IκBα (Ser32/36), β-actin and ubiquitin were from Cell Signaling Technology (USA). The antibodies against IκBα (ab47449) and RIP2 (ab8427) were from Abcam (UK).

### Fish cell culture

The rainbow trout hepatoma cell line RTH-149 was purchased from the American Type Culture Collection (ATCC, USA). Cells were cultured in Eagle’s minimal essential medium (EMEM), supplemented with non-essential amino acids, 10% foetal bovine serum (FBS), 2 mM L-glutamine, 1 mM sodium pyruvate, 25 mM HEPES, 10,000 units/ml penicillin and 10 mg/ml streptomycin. Cells were grown at 19 °C in the absence of CO_2_. Trypsin-EDTA (0.05%) was used to detach cells for subculturing. All the cell culture media and reagents were purchased from Lonza (Switzerland).

### RNA extraction, cDNA synthesis and quantitative real-time PCR

To examine the variation in OmPGRP-L1 expression in RTH-149 cells following stimulation with various ligands (PGN, LPS, LTA, iE-DAP and MDP), 1 × 10^6^ RTH-149 cells per well were seeded in 6-well plates and stimulated with different concentrations of each ligand for the indicated times. Total RNA was then extracted using an RNeasy kit (Qiagen, Germany) according to the manufacturer’s instructions. A total of 1 μg of RNA from each sample was reverse-transcribed with a Power cDNA synthesis kit (Intron, Korea) at 42 °C with oligo (dT)_15_ primers. Quantitative real-time PCR (qRT-PCR) was performed using SsoFast^TM^ EvaGreen Supermix (Bio-Rad Laboratories, Inc., USA) as previously described^13^. All of the primer sets used in the qRT-PCR are listed in Supplementary Table S1. The differences between groups were analysed using a Student’s *t*-test with GraphPad Prism version 5.00 (GraphPad Software, USA). Differences were considered significant at *P* < 0.05.

### Silencing of OmPGRP-L1, NOD1, NOD2 and A20 expression in RTH-149 cells

siRNA sequences targeting OmPGRP-L1, NOD1, NOD2, A20 and non-targeting negative control sequences were synthesised at Genolution (Korea). The siRNA sequences are shown in Supplementary Table S2. RTH-149 cells (1 × 10^5^ cells/well in 6-well plates) at 40% confluence were transfected with each siRNA (60 pmol) using the Lipofectamine 2000 transfection reagent (Invitrogen, USA). After 48 h, cells (except control cells) were stimulated with 10 μg/ml iE-DAP and 100 μg/ml MDP for 24 h and collected for the analysis of target gene expression using qRT-PCR.

### Inhibitor assay

To examine the variation in OmPGRP-L1 expression in iE-DAP- and MDP-stimulated RTH-149 cells following the suppression of NF-κB and RIP2 activity, 1 × 10^6^ RTH-149 cells per well were seeded in 6-well plates and pretreated with or without 25 μM NF-κB inhibitors (PDTC and TPCK) or 10 μM RIP2 inhibitor (gefitinib) for 30 min. After incubation, cells were stimulated for 8 h in the absence or presence of 100 μg/ml iE-DAP and 500 μg/ml MDP and collected for analysis of OmPGRP-L1 expression using qRT-PCR.

### Isolation and *in silico* analysis of the OmPGRP-L1 gene promoter region

Genomic DNA was extracted from RTH-149 cells using an i-genomic CTB DNA extraction mini kit (Intron) according to the manufacturer’s instructions. To allow chromosome walking beyond the known OmPGRP-L1 sequence into the unknown 5′ flaking promoter region, TAIL-PCR was performed using a DNA Walking SpeedUp Premix Kit (Seegene, Korea)^23^. Based on the cDNA sequence of the OmPGRP-L1 gene (GenBank Accession number JQ890076), a total of three gene-specific primers (GSP1, GSP2 and GSP3) in nested positions close to the 5′-end of the coding region were designed and synthesised. Three rounds of PCR were completed using the product of the previous PCR as a template for the next and employing a common arbitrary primer DW-ACP (provided in the kit) and nested gene-specific primers (GSP1, GSP2 and GSP3) in a consecutive manner. The primer sequences and reaction parameters for the TAIL-PCR are shown in Supplementary Tables S3 and S4. The amplified DNA fragment was cloned into a pGEM-T easy vector (Promega, USA), and the selected clone was sequenced in both directions using T7 and SP6 primers. The predictions of putative transcription factor binding sites in the promoter region of the OmPGRP-L1 gene were achieved with an *in silico* analysis using the TRANSFAC Professional Database (www.biobase.de/pages/products/transfac.html) and CONSITE (http://consite.genereg.net/cgi-bin/consite). A core similarity of 0.85 or greater and a matrix similarity of 0.90 or greater were defined as a cut-off for potential query sequence matches.

### Reporter plasmid construction and luciferase assay

The OmPGRP-L1 gene promoter, −1605 to −1 bp upstream of the start codon, was amplified by PCR from genomic DNA and cloned into the *Kpn*I and *Xho*I sites of the luciferase reporter vector pGL3 basic (Promega). The resultant vector was named P1(-1605)Luc. All deletion constructs, which have the same 3′ end but different 5′ ends, were made using P1(-1605)Luc as the PCR template and the same reverse primer used for the original construct. Restriction sites for *Kpn*I and *Xho*I were added to all upstream and downstream PCR primers, respectively. All constructs were analysed by restriction digestion and sequencing. Primers used for PCR are listed in Supplementary Table S3.

For the luciferase assay, approximately 1 × 10^5^ RTH-149 cells cultured in 24-well plates were co-transfected with a total of 1 μg of reporter plasmid DNA [P1(-1605)Luc, P1(-920)Luc, P1(-755)Luc, P1(-662)Luc, P1(-555)Luc or P1(-473)Luc] or the pGL3 basic vector with 50 ng of the pRL-TK vector (Promega) using the Lipofectamine 2000 reagent. At 48 h after transfection, cells (except control cells) were stimulated with 10 μg/ml iE-DAP and 100 μg/ml MDP for 24 h. Next, cells were lysed in lysis buffer (Promega) and assayed for firefly and Renilla luciferase activities. Firefly luciferase activity was normalised with that of Renilla activity. The results were obtained from three independent experiments performed in triplicate.

### EMSA

RTH-149 cells were cultured at 1 × 10^6^ cells/well in 6-well plates for 24 h. After incubation, cells were stimulated for 24 h in the absence or presence of 10 μg/ml iE-DAP and 100 μg/ml MDP. The cells were harvested, and nuclear proteins were extracted from cells using NE-PER Nuclear and Cytoplasmic Extraction Reagents (Pierce, USA). EMSA was performed using a LightShift chemiluminescent EMSA kit (Pierce). DNA oligonucleotides that included the predicted putative binding sites for NF-κB (κB_−691_ and κB_−496_) were ordered as single-stranded 3′-ends unlabelled or labelled with biotin from Genotech (Korea). The double-strand probes were prepared by annealing the 3′-end biotin-labelled and unlabelled oligonucleotides. The DNA-protein binding assay was performed at 22 °C for 30 min in a final volume of 20 μl containing 10 mM Tris (pH 7.5), 50 mM KCl, 10 mM DTT, 7.5% glycerol, 5 mM EDTA, 0.25% NP-40, 50 pg/ml Poly(dI·dC), 1 fmol of double-stranded biotinylated probe and 10 μg of the nuclear extract. To determine binding specificity, 100 × (0.1 pmol) of unlabelled specific oligonucleotides or non-specific oligonucleotides (mutant κB_−691_ and mutant κB_−496_) were included in the binding reaction. All samples were separated with 5% PAGE, and the DNA-protein complexes were visualised with the SuperSignal chemiluminescence reagent (Pierce). The oligonucleotides used for the EMSA are listed in Supplementary Table S5.

### OmPGRP-L1 overexpression in RTH-149 cells

An open reading frame (ORF) of OmPGRP-L1 was amplified from the OmPGRP-L1 full-length cDNA clone by PCR with a gene-specific primer set (PE-1F/PE-1R). The PCR fragment was digested with *Kpn*I and *Xho*I (restriction sites underlined in the primers) and then subcloned into pcDNA3.1 (Invitrogen) digested with the same restriction enzymes. The resultant vector was named pcDNA3.1-OmPGRP-L1-FLAG. RTH-149 cells were transfected with pcDNA3.1-OmPGRP-L1-FLAG or an empty pcDNA3.1 vector using the Lipofectamine 2000 transfection reagent, according to the manufacturer’s instructions. OmPGRP-L1 overexpression was confirmed with western blotting using a mouse monoclonal anti-FLAG antibody (1:500, Cell Signaling Technologies, USA). At 48 h after transfection, cells (except control cells) were stimulated with 10 μg/ml iE-DAP and 100 μg/ml MDP for 24 h and collected for the analysis of target gene expression using qRT-PCR.

### Preparation of conditioned media and media exchange experiments

RTH-149 cells (1 × 10^6^ cells/well in 6-well plates) at 90% confluence were transfected with pcDNA3.1-OmPGRP-L1-FLAG (OmPGRP-L1) or an empty pcDNA3.1 vector (empty vector) using the Lipofectamine 2000 transfection reagent, according to the manufacturer’s instructions. At 48 h after transfection, the medium was recovered. The supernatants, after a brief spin, were used as conditioned media (OmPGRP-L1 CM and empty vector CM, respectively). The secreted OmPGRP-L1-FLAG recombinant protein in the conditioned media was confirmed with western blotting using a mouse monoclonal anti-FLAG antibody.

To determine the effect of conditioned media on the production of NOD-mediated pro-inflammatory cytokines and NF-κB activation, media exchange experiments were performed. RTH-149 cells were seeded in 6-well plates at a density of 1 × 10^6^ cells/well, and the cell culture medium was replaced with the conditioned media containing the secreted OmPGRP-L1-FLAG recombinant protein (OmPGRP-L1 CM) or empty vector CM. Next, cells were stimulated with 10 μg/ml iE-DAP and 100 μg/ml MDP for 24 h and collected for the analysis of target gene expression using qRT-PCR. For the NF-κB assay, approximately 1 × 10^5^ RTH-149 cells cultured in 24-well plates were co-transfected with a total of 1 μg of pNF-κB-Luc (Clontech Laboratories Inc., USA) with 50 ng of the pRL-TK vector using the Lipofectamine 2000 reagent. At 48 h after transfection, the cell culture medium was replaced with the conditioned media. Next, cells were stimulated with 10 μg/ml iE-DAP and 100 μg/ml MDP for 24 h and collected for the measurement of NF-κB activity using a luciferase assay.

### Assays of endogenous protein ubiquitylation

RTH-149 cells (2 × 10^6^ cells/plate in 60 mm plates) at 90% confluence were transfected with pcDNA3.1-OmPGRP-L1-FLAG or an empty pcDNA3.1 vector using the Lipofectamine 2000 transfection reagent, according to the manufacturer’s instructions. At 48 h after transfection, cells (except control cells) were stimulated with 10 μg/ml iE-DAP and 100 μg/ml MDP for 24 h, lysed in RIPA lysis buffer, and immunoprecipitated with anti-RIP2 antibody. The immunoprecipitated polyubiquitylated proteins were subjected to 12% SDS-PAGE, and western blotting was performed using an anti-ubiquitin antibody. To ensure that equivalent amounts of RIP2 were immunoprecipitated, the immunoprecipitates were treated with 5% β-mercaptoethanol before SDS-PAGE, and analysed by immunoblotting with anti-RIP2 antibody.

## Additional Information

**How to cite this article**: Jang, J. H. *et al*. PGRP negatively regulates NOD-mediated cytokine production in rainbow trout liver cells. *Sci. Rep.* **6**, 39344; doi: 10.1038/srep39344 (2016).

**Publisher's note:** Springer Nature remains neutral with regard to jurisdictional claims in published maps and institutional affiliations.

## Supplementary Material

Supplementary Information
