# Supplementary material for: PGRP negatively regulates NOD-mediated cytokine production in rainbow trout liver cells
Source: Sci Rep. 2016 Dec 19;6:39344. doi: 10.1038/srep39344 (PMC5171823; doi:10.1038/srep39344)
Supplement: Supplementary Information [file srep39344-s1.pdf]

Supplementary Information for:

PGRP negatively regulates NOD-mediated cytokine production in rainbow trout liver cells

Ju Hye Jang[1], Hyun Kim[2], Mi Jung Jang[2], Ju Hyun Cho[1,2,*]

[1]Research Institute of Life Science, Gyeongsang National University, Jinju 52828, South Korea
[2]Division of Life Science, Gyeongsang National University, Jinju 52828, South Korea

[*]Corresponding author:
J.H. Cho
Division of Life Science, Gyeongsang National University, 501 Jinju Dae-ro, Jinju 52828, South Korea
Phone: 82-55-772-1347. Fax: 82-55-772-1349. E-mail address: juhyun.cho@gnu.ac.kr

Includes 5 tables and 1 figure.

Supplementary Table S1. List of primer sequences used for qRT-PCR.

Gene name	Forward primer (F)<br>Reverse primer (R)	Sequence	Length (nt)	Accession no.
OmPGRP-L1	F	5′-GTG GTC TTG GTC AGT GCT CAC ACT-3′	24	JQ890076
OmPGRP-L1	R	5′-CTG CGC AGG CCT CTC AAC ACA TTT-3′	24	JQ890076
NOD1	F	5′-CAT CTA TCT TTG ACG TGG GAA TG-3′	23	KF484402
NOD1	R	5′-GAG GCT GAG GTT GGT AAG AC-3′	20	KF484402
NOD2	F	5′-TCT CTC TCT AAG GCT GGG AAA C-3′	22	NM_001201555
NOD2	R	5′-TTG CCA ACA CCA TTG TCT ACC A-3′	22	NM_001201555
IL-1β	F	5′-ACA TTG CCA ACC TCA TCA TCG-3′	21	AJ223954
IL-1β	R	5′-TTG AGC AGG TCC TTG TCC TTG-3′	21	AJ223954
IL-6	F	5′-CCT TGC GGA ACC AAC AGT TTG-3′	21	DQ866150
IL-6	R	5′-CCT CAG CAA CCT TCA TAT GGT C-3′	22	DQ866150
IL-8	F	5′-AGA ATG TCA GCC AGC CTT GT-3′	20	AJ279069
IL-8	R	5′-TCT CAG ACT CAT CCC CTC AGT-3′	21	AJ279069
TNF-α	F	5′-GGG GAC AAA CTG TGG ACT GA-3′	20	AJ277604
TNF-α	R	5′-GAA GTT CTT GCC CTG CTC TG-3′	20	AJ277604
A20	F	5′-TGT TCA GCG TGC TAA AGG AGA CAG-3′	24	DQ400414.1
A20	R	5′-ATC TGC GAT GAC GAT GAT GGG C-3′	22	DQ400414.1
β-actin	F	5′-GCG GGC CGC GAC CTC ACA GAC TAC-3′	24	NM_001124235
β-actin	R	5′-CGG CCG TGG TGG TGA AGC TGT AAC-3′	24	NM_001124235

Supplementary Table S2. List of siRNA sequences used in the study.

Target protein	Sequence
OmPGRP-L1	Sense: 5′-CCU CAG AGG CAC CAG UUA UUU-3′<br>Antisense: 5′-AUA ACU GGU GCC UCU GAG GUU-3′
NOD1	Sense: 5′-GGG ACA UAC CAG UGA AGA AUU-3′<br>Antisense: 5′-UUC UUC ACU GGU AUG UCC CUU-3′
NOD2	Sense: 5′-CCA GAC CAU CAC AUA CGU UUU-3′<br>Antisense: 5′-AAC GUA UGU GAU GGU CUG GUU-3′
A20	Sense: 5′-CGU UAC AGC ACC AUG AAU UGG UU-3′<br>Antisense: 5′-CCA AUU CAU GGU GCU GUA ACG UU-3′
Scrambled siRNA of OmPGRP-L1	Sense: 5′-GCC AUG ACC AUA CGU AGC UUU-3′<br>Antisense: 5′-AGC UAC GUA UGG UCA UGG CUU-3′
Non-specific siRNA of NODs and A20	Sense: 5′-AGA UCC GCU ACU GUC CGA AUU-3′<br>Antisense: 5′-UUC GGA CAG UAG CGG AUC UUU-3′

Supplementary Table S3. List of primer sequences used for cloning.

Primer name	Sequence	Length (nt)
PE-1F	5′-GGT ACC ATG TCA GGA GCA CAG GGC-3′	24
PE-1R	5′-CTC GAG TCA CTT ATC GTC GTC ATC CTT GTA ATC GTT TTG AAC CTC CCC AAA G-3′	52
GSP1	5′-CTT CTT CCT CTA AGT AGC TGT AAT GC-3′	26
GSP2	5′-TCA GTG TGA GCA CTG ACC AAG ACC AC-3′	26
GSP3	5′-CAT TGA GTC TTC GAC GCC CTG TGC TC-3′	26
P1(-1605)F	5′-GGT ACC GTC GGA CAC AGA GTA CAC G-3′	25
P1(-1235)F	5′-GGT ACC TAA CAA GTG CCT ATG GTG GTA GTA TG-3′	32
P1(-1190)F	5′-GGT ACC CGT CAC AGC TCG CTG TGT A-3′	25
P1(-920)F	5′-GGT ACC GCC AGC TAA TGT AGC TGC-3′	24
P1(-755)F	5′-GGT ACC CAA GGA CCT AAA ACC ACT TAC C-3′	28
P1(-662)F	5′-GGT ACC GCA CTG ATC TGT GAA CAG C-3′	25
P1(-555)F	5′-GGT ACC CCT AAT GTC AAT TGC ATG GTT AAA-3′	30
P1(-1)R	5′-CTC GAG GTA TTA CGC ATT GCA TCG CC-3′	26

*Kpn*I and *Xho*I restriction sites are underlined, and the FLAG sequence is double-underlined in the primers.

Supplementary Table S4. Reaction parameters for the TAIL-PCR used to amplify the 5′-flanking regions of the OmPGRP-L1 gene.

Reaction (primer combination)	Template	Number Of cycles	Cycle parameters
Primary PCR (DW-ACP/GSP1)	Genomic DNA	1	94 °C, 5 min; 42 °C, 1 min; 72 °C, 2 min
		30	94 °C, 40 s; 55 °C, 40 s; 72 °C, 1 min
		1	72 °C, 7 min
Secondary PCR (DW-ACP/GSP2)	Product of primary PCR	1	94 °C, 5 min
		35	94 °C, 40 s; 60 °C, 40 s; 72 °C, 1 min
		1	72 °C, 7 min
Tertiary PCR (DW-ACP/GSP3)	Product of secondary PCR	1	94 °C, 5 min
		30	94 °C, 40 s; 65 °C, 40 s; 72 °C, 1 min
		1	72 °C, 7 min

Supplementary Table S5. List of oligonucleotide sequences used for EMSA.

Name	Sequence
κB$_{-691}$	Sense: 5′-TTG TGT TGG GGA CTT TCC TGT T-3′<br>Antisense: 5′-AAC AGG AAA GTC CCC AAC ACA A-3′
κB$_{-496}$	Sense: 5′-ATG CAT GGG AAT TTC CTT TGA G-3′<br>Antisense: 5′-CTC AAA GGA AAT TCC CAT GCA T-3′
Mutant κB$_{-691}$	Sense: 5′-TTG TGT TGG TGA CTG TAC TGT T-3′<br>Antisense: 5′-AAC AGT ACA GTC ACC AAC ACA A-3′
Mutant κB$_{-496}$	Sense: 5′-ATG CAT GAG TAT GTA CTT TGA G-3′<br>Antisense: 5′-CTC AAA GTA CAT ACT CAT GCA T-3′

```
-1635 TCACAGAAGTATGCCAAGCGAGGGGGGGGGGTCGGACACAGAGTACACGCGGCTCTGGCAGTACTGCACCATCTCAGAGAAGAGCAGTGCAAAGGCACTC -1536

-1535 ACACTGACTTCAGTCTTGGGACGGGTTAGAGGCCGTTCCAGGATGTTTGACTTTCCTCTCGTGAACCGAGTGTCCATTTTCTGAAAGAGAATGATCTGTA -1436
           AP-1                                   NF-κB
-1435 AATAGAAGTAAAATAGACTGAAGTGATAATATGCTAGCCATGTCAATTCCAAATCAACCCCTCACCACTACCCTAGGCAGGTGAAGATCTGAGAGGATTA -1336

-1335 GCTATAGATAGATAACACTTTCAATTAGCCTAGGGGGAAGGATCTTCAACCACAATGTTTAGCTATCCGTCTTTTAACAAGGTAGTATTTTTTTAGATCT -1236

-1235 TAACAAGTGCCTATGGTGGTAGTATGGCTCACACCTCTCGAATTCGTCACAGCTCGCTGTGTAGTCACGTGCCTCGCGCGTAAATCAGGTGAAAGGGGTA -1136
                                AP-1                              AP-1
-1135 GGGCCTATGGGGGTTGTTTTGGGATTCAGGTCGCCGCGCCTCAAGAGGGCCAGCTTCGGTTGTGGCAATCTTTACCTGCAGTACATATGCAAAGACATTA -1036

-1035 GCCAACGACGATTCCGTGAATTAGGAGGAGCTAACTGCGTTGAAAAAACAACAAAGTCGCTGCTAGTGCAGACGAAAGTGGGAAGATAAAGTATATTGGA -936

 -935 TGCTAGCGTAACTAGCCAGCTAATGTAGCTGCTAGCTAGTGCAAATTTAGTTAATATTGTCAAGCAAGCTAGCGAACTCACTCGACGACAGAAAGGCCAG -836

 -835 AACTCAGTCATTCAGAATGTTAACAAGCTAACATTTAAACATATTTATTTATTTATTTATATAGTTCATTATTCTTATACAAGGACCTAAAACCACTTAC -736
         AP-1
 -735 CGCGCTTCAATGTTTTTAGCCTGCTGTTGTGTTGGGGACTTCCTGTTCTGGTCACGTGTCTTGTTCTAGTAAAGCACTGATCTGTGAACACCAGTGCATG -636
                                          NF-κB
 -635 TGCTCATCTGACACATACCTGTTTCTACATGTGTAATCTAAATTACATGCTTTGAAAAAGTTTTAAAAAAATGCATGATTTCCTAATGTCAATTGCATGGT -536
             AP-1
 -535 TAAAAAAAATATTCCTGTTAAATGATGCATGGGAATTTCCTTTGAGTTCTTGCTGTGTATTTCTAGGGCCATTTGGAAAAGGAACAAGTACATGCTTGCA -436
                                     NF-κB
 -435 TTCTCATGCGTCACATACCTTTCAGTGATTTAAAAGATACTGTTATCCAAACACAACATACAATATGATGCTGCTGTACAGTACTTTCTAACAGAATTTT -336
           AP-1            AP-1
 -335 GTTTAATATTGAGATATTATGAAACAACAGTCTATACAAAGTACTTGAGGAGAAGGGGAAAATAAATTGCAGACCTAAAGGTTGTACTTGTGACAGTGGT -236

 -235 GTAATGCAATAATGTAAATAATGAAATGTTTAAATTCAGAAAGACCTTGAGCCATAAGGACAAACACACATGTACACACACACACTTACCTAACAGTTGC -136
                                                     AP-1
 -135 CAATTTCCCACACCATGTCCCTCAAATGACTCAAATTAACCAGCACCGTATCTGTTTGTACAGTGGTTGCATAATCCACTTGATGTGTTTGCCTTCCTAT -36
                                AP-1
                 *
  -35 AAATGTTTCAAGGTAGGCGATGCAATGCGTAATACATGTCAGGAGCACAGGGCGTCGAAGACTCAATGATTTCTCCAATGGAACCGCACTGGAAATGGTG +65
                                     +1
```

Supplementary figure S1. Nucleotide sequence of the OmPGRP-L1 gene promoter. The nucleotide sequence of the upstream region of the OmPGRP-L1 gene is shown. Nucleotides are numbered relative to the start codon with A of ATG (underlined bold) being +1. The 5′ end of OmPGRP-L1 mRNA identified from the OmPGRP-L1 cDNA sequence is represented by an asterisk and bold letter. Potential NF-κB and AP-1 binding sites are shown by a line below the sequence and by the name of the transcription factor.
